# Maternal and neonatal glycaemic control after antenatal corticosteroid administration in women with diabetes in pregnancy: A retrospective cohort study

**DOI:** 10.1371/journal.pone.0246175

**Published:** 2021-02-18

**Authors:** Jeremy F. Tuohy, Frank H. Bloomfield, Caroline A. Crowther, Jane E. Harding

**Affiliations:** Liggins Institute, University of Auckland, Auckland, New Zealand; Mount Sinai Health System, University of Toronto, CANADA

## Abstract

**Objective:**

To describe maternal and neonatal glycaemic control following antenatal corticosteroid administration to women with diabetes in pregnancy.

**Design:**

Retrospective cohort study

**Setting:**

A tertiary hospital in Auckland, New Zealand

**Population:**

Women with diabetes in pregnancy who received antenatal corticosteroids from 2006–2016.

**Methods:**

Corticosteroid administration, maternal and neonatal glycaemia data were retrieved from electronic patient records. Demographic data were downloaded from the hospital database. Relationships between variables were analysed using multivariate analysis.

**Main outcome measures:**

Maternal hyperglycaemia and neonatal hypoglycaemia

**Results:**

Corticosteroids were administered to 647 of 7317 of women with diabetes (8.8%) who gave birth to 715 babies. After an initial course of corticosteroids, 92% and 52% of women had blood glucose concentrations > 7 and > 10 mmol/L respectively. Median peak blood glucose concentration of approximately 10 mmol/L occurred 9 hours after corticosteroid administration and hyperglycaemia lasted approximately 72 hours. Thirty percent of women gave birth within 72 hours of the last dose of corticosteroids. Babies of women who were hyperglycaemic within 24 hours of birth were more likely to develop hypoglycaemia (< 2.6 mmol/L, OR 1.51 [95% CI 1.10–2.07], p = 0.01) and severe hypoglycaemia (≤ 2.0 mmol/L, OR 2.00 [95% CI 1.41–2.85], p < 0.0001) than babies of non-hyperglycaemic mothers. There was no association between maternal glycaemia within 7 days of the last dose of corticosteroids and neonatal hypoglycaemia.

**Conclusions:**

Hyperglycaemia is common in women with diabetes in pregnancy following antenatal corticosteroid administration. Maternal hyperglycaemia in the 24 hours prior to birth is associated with increased risk of neonatal hypoglycaemia. Limitations included the retrospective study design, so that not all data were available for all women and babies and the glucose testing schedule was variable.

## Introduction

Antenatal corticosteroids (ANC) improve outcomes for babies born preterm [1] but cause maternal hyperglycaemia [2] particularly in women with diabetes in pregnancy (DIP) [2–4]. Limited data suggest that maternal blood glucose concentration (BGC) is elevated for at least 48 hours after two doses of ANC [5, 6] although this time period is uncertain for women with DIP. The recommended optimal time for ANC administration prior to elective birth is 48 hours [7]. As approximately 25% of all women who receive ANC give birth within 24 hours [8], maternal hyperglycaemia is likely to peak close to the time of birth for many women.

Maternal blood glucose crosses the placenta by facilitated diffusion. Thus, maternal hyperglycaemia leads to fetal hyperglycaemia and increased insulin secretion [9] which, if persistent, can result in neonatal hypoglycaemia. In women without diabetes, a high glucose load given prior to elective caesarean section led to maternal hyperglycaemia and neonatal hypoglycaemia [10]. A recent systematic review of 23 studies in women with diabetes found that 11 identified an association between maternal hyperglycaemia and neonatal hypoglycaemia [11]. However, there was considerable heterogeneity amongst studies, with multiple definitions used for both maternal hyperglycaemia and neonatal hypoglycaemia. In both women with type-1 diabetes [12] and with gestational diabetes (GDM) [4], continuous glucose monitoring during pregnancy has been shown to improve maternal glycaemic control and decrease the rate of neonatal complications including neonatal hypoglycaemia. Algorithms for insulin administration after ANC administration also have been shown to improve maternal glycaemic control [13, 14] and decrease the rate of neonatal hypoglycaemia [14].

Babies born to women with DIP are more likely to be preterm and be exposed to ANC than babies born to mothers without diabetes [15]. Approximately 50% of newborns of mothers with DIP become hypoglycaemic [16], which is associated with adverse neurodevelopmental outcomes [17]. The rate of neonatal hypoglycaemia has been reported to be increased in babies exposed to ANC prior to both preterm birth [2, 18] and following elective caesarean section at term [19]. A randomised controlled trial [20] found a higher incidence of hypoglycaemia in babies born late-preterm to women randomised to ANC compared with placebo. However, the relationship between ANC, maternal hyperglycaemia and neonatal hypoglycaemia remains unclear.

We aimed to describe, in a cohort of women with DIP receiving ANC, the rate of maternal hyperglycaemia and its temporal relationship to ANC, the rate of neonatal hypoglycaemia, and the relationship between maternal hyperglycaemia and neonatal hypoglycaemia.

## Methods

### Study design, participants and setting

We undertook a retrospective cohort study of women who had DIP (type 1, type 2 or gestational diabetes) birthing at National Women’s Health, Auckland, New Zealand, a tertiary obstetric referral hospital, between 1 January 2006 and 31 December 2016. ANC exposure was defined as any corticosteroid administration for the purpose of improving neonatal outcome from 22 completed weeks of pregnancy.

### Data collection

Maternal medical records were reviewed for all women giving birth at < 37^0^ weeks’ gestation; admitted to hospital for > 2 hours for any reason during pregnancy between 22^0^−36^6^ weeks’ gestation; birthing by elective caesarean section at any gestation; or birthing by emergency caesarean section at < 39 weeks’ gestation. All babies born to included mothers were identified from the hospital database. Details of ANC administration (drug, dose, date and time of each dose) were retrieved from prescription records. The indication for ANC administration was not available. An initial course of ANC was defined as the first course of ANC administered in the pregnancy. A repeat course of ANC was defined as a second or later course of ANC administered 7 days or more after the initial course. Maternal demographic, obstetric and neonatal data were extracted from the hospital database and entered into a secure anonymised research database. Further details of the cohort have been described previously [21].

All available maternal and neonatal BGCs, measured both by laboratory and point-of-care devices, including in-patient, outpatient and self-recorded measurements, were obtained from the clinical records. For mothers, all BGCs from 48 hours prior to the first dose of ANC until 7 days after the last dose for each course, or until the time of birth if this occurred before 7 days. BGCs were also collected for the 24 hours prior to birth. BGCs measured before breakfast were assumed to be fasting. For babies, all BGCs from birth until 7 days after birth. Insulin data were collected from hospital records and also patient-held records where available.

### Definitions

Definitions used for maternal glycaemic control for each period were: hypoglycaemia, < 3.5 mmol/L; elevated fasting BGC, ≥ 5.5 mmol/L; hyperglycaemia, BGC above a threshold of 7, 8, 10 or 11 mmol/L; peak hyperglycaemia, maximum BGC; time to onset of hyperglycaemia, time between administration of the first dose of ANC and the first BGC above the threshold; time to resolution of hyperglycaemia, the time from the first dose of ANC to the last hyperglycaemic recording, and blood glucose measurements out of glycaemic target range, < 4.0 mmol/L or > 7.0 mmol/L.

Definitions used for neonatal glycaemic control were: neonatal hypoglycaemia, < 2.6 mmol/L; severe hypoglycaemia, ≤ 2.0 mmol/L [22]; recurrent hypoglycaemia, a second or subsequent BGC of ≤ 2.6 mmol/L occurring after resolution of a previous episode of hypoglycaemia (defined as at least one BGC of > 2.6 mmol/L); hyperglycaemia, > 8.0 mmol/L [23].

### Clinical management

Hospital guidelines for the administration of ANC did not recommend administration prior to elective caesarean section. Guidelines for management of maternal hyperglycaemia did not change during the study period and recommended maintaining BGC between 3.5 and 7 mmol/L. Insulin administration was recommended after ANC administration if the BGC exceeded 7 mmol/l on two occasions or exceeded 8 mmol/l once [24]. Hospital guidelines for screening for neonatal hypoglycaemia recommended that all neonates born to mothers with DIP had BGC measured within 1–2 hours of birth, then four hourly until the baby was feeding well and three consecutive measurements were ≥ 2.6 mmol/l [25]. Use of oral dextrose gel for treatment of neonatal hypoglycaemia was introduced in late 2013.

### Analysis

Clinical information from the maternity database was imported into a study database created using JMP-14 (SAS, Cary, North Carolina). Details from the clinical record, including variables missing from the maternity database, were transcribed into the database. A review of 10% of the records by an independent researcher identified fewer than 1% of checked records with discrepancies in ANC, maternal or neonatal BGC data. Outlying data points were not removed. Mothers and babies with no glycaemic data were excluded from analysis. We did not impute any values for missing data.

The time course of maternal BGC was described by calculating the proportion of women exceeding each threshold and the mean BGC in each consecutive three-hour epoch after ANC administration. For women who had received insulin prior to ANC administration, the increase in insulin dose after ANC was calculated as the percentage increase in the 24 hour period after ANC administration compared to that in the 24 hour period immediately prior to ANC administration. For women who had not received insulin prior to ANC administration, the administration of insulin over the subsequent 24 hours was recorded as “yes” or “no”.

Data are presented as number and %, number and interquartile range (IQR) or range, and mean and standard deviation (SD). Maternal, neonatal and ANC variables were compared using nominal logistic regression and results presented as proportions with odds ratios and 95% confidence intervals. The results from the univariate analyses were used to construct multivariate models using multinomial logistic forwards and backwards stepwise regression. Results from the multinomial logistic regression are presented unless otherwise specified. A two-sided p value of < 0.05 was considered significant. Associations between maternal and neonatal glycaemia were analysed for each mother and baby pair, with multiple pregnancies treated as separate pairs. Using a nested analysis or using only one of the mother baby pairs did not change any of the results.

### Ethics

The Northern B Health and Disability Ethics Committee approved this study, including access to patient records without informed consent (Reference 16NTB216).

## Results

Details of the study cohort have been reported previously [21]. In brief, 7317 women with DIP were identified from the hospital database, of whom 647 (8.8%) received ANC. Of these, 234 (3.2%) had no blood glucose data at the time of ANC administration or within 24 hours of birth. Glycaemic data were available for 556 women after an initial course, 585 after the last course and 579 within 24 hours of birth. The cohort was approximately 25% European, 16–20% each Asian, Indian and Pacific and 10% each for Māori and ‘other’ (Table 1). Sixty-five percent had gestational diabetes, 21% type-2 and 13% type-1 diabetes, and 47% had a BMI > 30 kg/m^2^. There were 63 (11%) women with a multiple pregnancy (61 twins and 2 triplets) and 417 (72%) of women birthed by caesarean section (Table 1).

**Table 1:** Characteristics of women with diabetes in pregnancy who received antenatal corticosteroids and their babies for whom glycaemic data were available.

Mothers	Initial course = 556		Last course = 585		Birth = 579	
**Ethnicity**	**n**	**%**	**n**	**%**	**n**	**%**
Asian	96	17	106	18	112	19
Indian	92	17	91	16	91	16
Māori	68	12	69	11	70	12
New Zealand European	140	25	151	26	145	25
Pacifica	111	20	118	20	114	20
Other	49	9	50	9	47	8
**Maternal age (years)**						
< 25	38	7	37	6	37	6
25–34	272	49	288	49	292	50
35 and more	246	44	260	44	250	43
**Birth year**						
2006–2009	178	32	185	32	182	31
2010–2013	233	42	246	42	245	42
2014–2016	145	26	154	26	152	26
**Parity**						
0	205	37	216	37	218	38
1	185	33	192	33	187	32
2 or more	166	30	177	30	174	30
**Body mass index (kg/m**^**2**^**)**						
< 20	21	4	22	4	20	3
20–24.9	133	24	147	25	143	25
25–29.9	141	25	145	25	145	25
30 or more	261	47	270	46	270	47
**Multiple pregnancy**						
Multiple	58	10	59	10	54	9
Singleton	498	90	526	90	525	91
**Type of diabetes**						
Gestational Diabetes	360	65	389	66	380	66
Type-1	77	14	76	13	77	13
Type-2	119	21	120	21	122	21
**Mode of birth**						
Caesarean Section—Elective	205	37	215	37	206	36
Caesarean Section—Emergency	186	33	202	35	200	35
Operative Vaginal Birth	34	6	32	5	36	6
Spontaneous Vaginal Birth	131	24	136	21	137	24
**Babies**	**N = 714**					
**Birth gestation (weeks)**	**N**		**%**			
< 29^0^	37		5			
29^0^–32^6^	152		21			
33^0^–34^6^	159		22			
35^0^–36^6^	177		25			
37^0^ or more	189		26			
**Birthweight (g)**						
< 1500	111		16			
1500–2500	280		39			
2501–4000	277		39			
> 4000	46		6			
**Birthweight Centile**						
< 3rd	28		4			
3-10th	56		8			
10-90th	493		69			
> 90th	137		19			
**Female**	321		45			
**Admitted to neonatal nursery**	464		65			

Overall, 74% of babies were born preterm and 65% admitted to the neonatal nursery. Only 5% of babies were born at < 29 weeks of gestation and approximately 25% each at 29–32, 33–34, 35–36, and ≥ 37 weeks’. Birthweight was > 90th centile for 19% of babies, between the tenth and 90th centile for 69%, < tenth centile for 12% and < third centile for 4%. Sixty-five percent were admitted to the neonatal nursery (Table 1).

For an initial course of ANC, 97% of women received 11.4 mg betamethasone, 85% received 2 doses at a 24-hour interval, 17% received their first dose at more than 34 weeks’ gestation, 23% received their last course of ANC at more than 34 weeks’ gestation and 18% received a repeat course of ANC, of which 92% comprised a single dose (S1 Table).

### Maternal glycaemic control

The median (IQR) number of BGC recordings during the study period was 17 (7–30) for the initial course, 15 (6–28) for the last course, 11 (3–26) for a repeat course and was 5 (3–7) in the 24 hours prior to birth. The median peak maternal BGC was similar after the initial, last and repeat courses of ANC (10.1, 9.9 and 9.2 mmol/L respectively) and was 6.8 mmol/L in the 24 hours prior to birth (S2 Table). Hypoglycaemia occurred in 22% of women before and 28% after the initial course of ANC. Women with type 1 diabetes had the highest number of BGC measurements (24 (17–42), the highest peak BGC (13.4 mmol/L) and the largest proportion who experienced hypoglycaemia both before (49%) and after (58%) ANC. (S2 Table).

For an initial course of ANC, 92% of women were hyperglycaemic at a threshold of 7 mmol/L, 83% at a threshold of 8 mmol/L, 52% at a threshold of 10 mmol/L and 35% at a threshold of 11 mmol/L (S3 Table). Similar proportions were observed after the last course, and for women with different types of diabetes (S3 Table).

**Fig 1:**
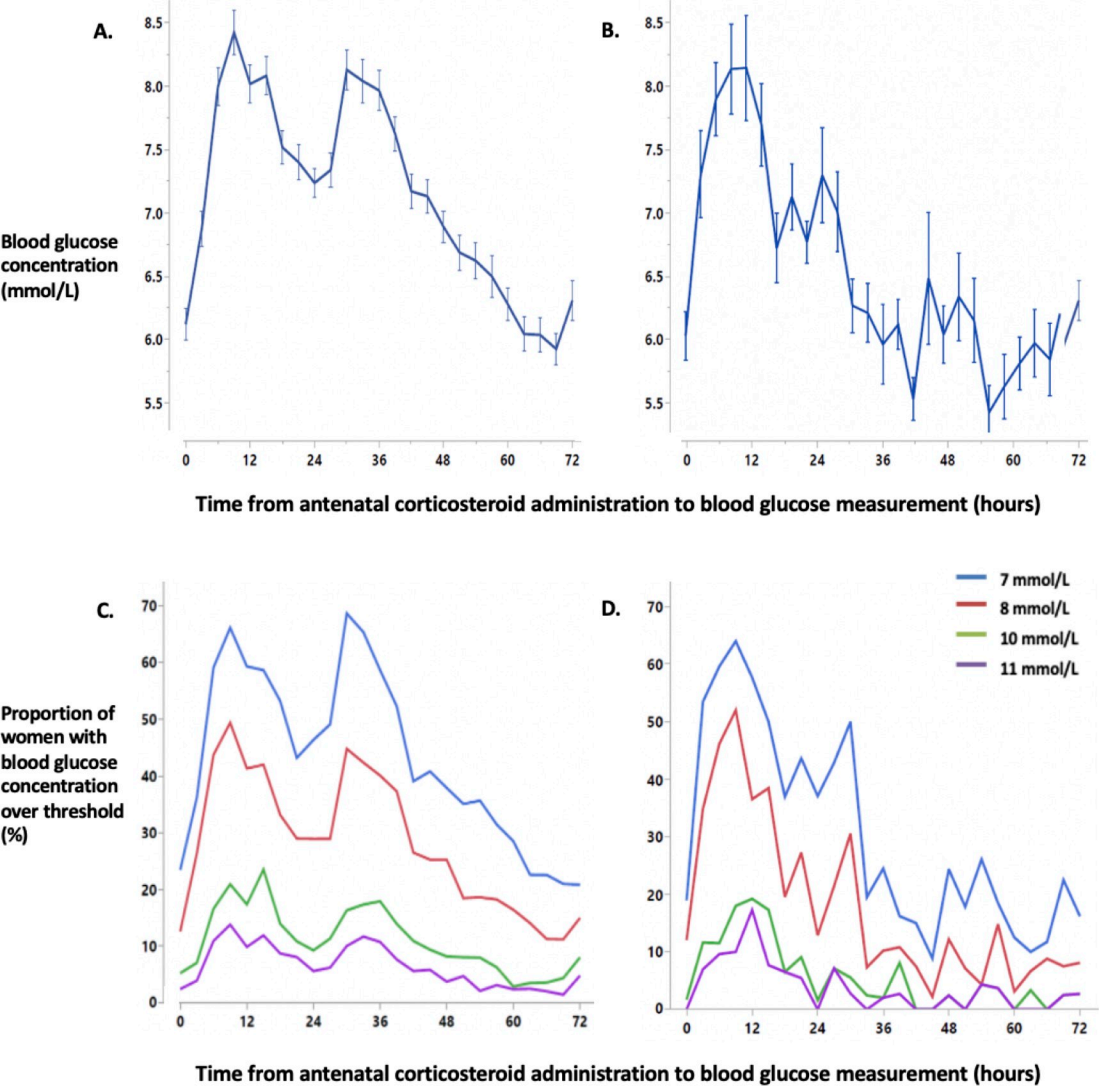
Mean blood glucose concentration and the percentage of women with a blood glucose measurement over threshold after an initial course of antenatal corticosteroids (panels A and C) and the last repeat course (panels B and D). Data for blood glucose concentration are mean with standard error. Blood glucose concentration data were analysed in 3-hour epochs. Time “0” represents blood glucose concentration values from three hours prior to the first dose of ANC administration in a course until antenatal corticosteroid administration. Panel A, mean blood glucose concentration after an initial course of ANC. Panel B, mean blood glucose concentration after the last repeat course of ANC. Panel C, the proportion of women with blood glucose over each hyperglycaemic threshold after the initial course of ANC. Panel D, the proportion of women with blood glucose over each hyperglycaemic threshold after the last repeat course of ANC.

Maternal BGC increased after the first dose of the initial course of ANC from a mean (SD) baseline of 6.1 (2.1) mmol/L, reaching a peak of 8.4 (2.7) mmol/L at 9 hours and falling to a nadir of 7.2 (2.1) mmol/L at 24 hours (Fig 1). A second peak with mean 8.1 (2.4) mmol/L occurred at 30 hours before BGC returned to pre-ANC levels at approximately 72 hours. After a repeat course of ANC, the pattern was similar, with second smaller peak of 7.1 (2.0) mmol/L at 27 hours. This second peak remained even when women receiving two doses of ANC for a repeat course were excluded. The proportion of women whose BGCs exceeded the different hyperglycaemic thresholds showed a similar pattern (Fig 1).

On multivariate analysis, pre-existing diabetes was the strongest independent predictor of maternal hyperglycaemia, with birth from 2010 onwards predictive of a significantly lower risk of hyperglycaemia compared with birth between 2006–2009 (S4 Table).

On univariate analysis, the rate of maternal hyperglycaemia at all thresholds was significantly increased for women who completed an initial course of ANC compared to those who did not and, at a threshold of 7 mmol/L, was increased for women who received two doses of ANC at a 24-hour interval compared to a 12-hour interval (Table 2). The median (IQR) duration of hyperglycaemia at a threshold of 8 mmol/L, but not other thresholds, was significantly less for women receiving ANC at a 12-hour compared to a 24-hour interval (13 (5–44) vs 33 (17–89) hours, p = 0.002). Women receiving an initial course of ANC after 34 weeks’ gestation were less likely to develop hyperglycaemia than those receiving ANC between 24–34 weeks’ gestation. Hyperglycaemia was less common after a repeat course of ANC than an initial course and women who received 1 dose of ANC for their last course were significantly less likely to develop hyperglycaemia than those receiving two doses. Women receiving either an initial or last course of ANC at more than 34 weeks’ gestation were less likely to develop hyperglycaemia than women receiving ANC at less than 35 weeks’ (Table 2).

**Table 2:** Relationships between antenatal corticosteroid variables and the rate of maternal hyperglycaemia.

**Univariate analysis**												
**ANC variable**					**Hyperglycaemic Threshold**							
	**7 mmol/L**			**8 mmol/L**			**10 mmol/L**			**11 mmol/L**		
	**OR**	**95% CI**	**p-value**	**OR**	**95% CI**	**p-value**	**OR**	**95% CI**	**p-value**	**OR**	**95% CI**	**p-value**
**Initial Course**												
**Complete course of ANC (2 doses)**												
** Yes**	**Reference**											
No	0.05	0.02–0.09	< 0.0001	0.11	0.06–0.20	< 0.0001	0.10	0.03–0.20	< 0.0001	0.17	0.06–0.49	0.001
**Schedule of initial course**												
** 24 hours**	**Reference**											
12 hours	0.18	0.06–0.52	0.002	0.80	0.29–2.16	0.66	0.56	0.27–1.18	0.13	0.96	0.44–2.04	0.91
**Gestation at administration of first dose of initial ANC course**												
** 24–34 weeks**	**Reference**											
≥ 35 weeks	0.38	0.19–0.75	0.003	0.45	0.28–0.76	0.002	0.58	0.35–0.96	0.036	0.56	0.34–0.92	0.027
**Last Course**												
**Repeat course of ANC**												
** No**	**Reference**											
Yes	0.66	0.33–1.32	0.25	0.51	0.31–0.84	0.008	0.42	0.26–0.67	0.0003	0.57	0.34–0.94	0.03
**Number of doses in last course**												
** 2**	**Reference**											
1	0.15	0.09–0.29	< 0.0001	0.24	0.15–0.37	< 0.0001	0.28	0.18–0.43	< 0.0001	0.40	0.25–0.64	0.0002
**Gestation at administration of first dose of last ANC Course**												
** 24–34 weeks**	**Reference**											
≥ 35 weeks	0.32	0.18–0.57	0.0001	0.49	0.32–0.77	0.002	0.59	0.41–0.86	0.006	0.67	0.44–1.03	0.07
**Multivariate analysis**												
**Initial Course**												
**Complete course of ANC (2 doses)**												
** Yes**	**Reference**											
No	0.04	0.02–0.09	< 0.0001	0.09	0.05–0.18	< 0.0001	0.10	0.04–0.27	< 0.0001	0.17	0.06–0.49	< 0.0001
**Schedule of initial course**												
** 24**	**Reference**											
12	0.23	0.08–0.62	0.004	0.69	0.31–1.52	0.36	0.57	0.27–1.21	0.15	0.97	0.45–2.09	0.95
1 Dose	0.06	0.03–0.13	< 0.0001	0.15	0.31–1.52	< 0.0001	0.10	0.04–0.26	< 0.0001	0.17	0.06–0.49	0.001
**Gestation at ANC administration**												
** 24–34**	**Reference**									
≥ 35	0.37	0.19–0.74	0.005	0.50	0.31–0.80	0.004	0.55	0.35–0.86	0.008	0.61	0.37–0.98	0.04
**Last Course**												
**Total number of doses in pregnancy**												
** 2 doses**	**Reference**											
1 dose	0.35	0.01–8.90	0.52	0.38	0.06–2.36	0.31	0.50	0.11–2.32	0.39	0.49	0.09–2.60	0.40
≥ 3 dose	1.76	0.07–40.0	0.74	1.02	0.19–5.47	0.97	0.96	0.28–3.22	0.95	0.80	0.24–3.21	0.84
**Schedule of last course**												
** 24**	**Reference**											
12	0.12	0.04–0.38	0.0003	0.52	0.18–1.49	0.22	0.58	0.24–1.39	0.22	0.84	0.33–2.11	0.71
1 dose	0.13	0.005–3.23	0.21	0.29	0.05–1.61	0.16	0.32	0.09–1.15	0.08	0.48	0.12–1.92	0.31
**Gestation at ANC administration**												
** 24–34**	**Reference**											
≥ 34	0.42	0.09–1.91	0.26	0.37	0.14–1.01	0.054	0.56	0.29–1.07	0.08	0.47	0.25–0.88	0.02

OR, odds ratio; ANC, antenatal corticosteroids.

On multivariate analysis including the completeness of the course of ANC, the time between doses and the gestation at administration, the significant associations between maternal hyperglycaemia and the completeness of the course and the gestation at administration remained for the initial course. However, the decreased risk of maternal hyperglycaemia after a 12-hour interval between doses compared to a 24-hour interval remained only for a threshold of 7 mmol/L (Table 2). After the last course of ANC, the risk of maternal hyperglycaemia at any threshold for women who had either one dose or three or more doses during the pregnancy, compared to only two doses was not significantly different. However, women receiving three or more doses of ANC were more likely to be hyperglycaemic than women receiving only one dose of ANC at a threshold of 7 mmol/L (OR 4.82, 95% CI 2.05–11.81, p = 0.0003) and 8 mmol/L (OR 2.73, 95% CI 1.34–5.66, p = 0.005) but not at higher thresholds (10 mmol/L: OR 2.21, 95% CI 0.93–5.89, p = 0.07; 11 mmol/L: OR 1.97, 95% CI 0.69–5.61, p = 0.21). Women who received a last course of ANC at more than 34 weeks’ gestation were less likely to be hyperglycaemic than women who received a last course before this gestation, with odds ratios around 0.5 for all BGC thresholds, although this was statistically significant only at a threshold of 11 mmol/L (Table 2).

The majority of women with pre-existing diabetes were receiving insulin prior to ANC administration (S5 Table). For women with gestational diabetes, the proportion receiving insulin increased from 40% before to 66% after ANC administration. Amongst women receiving insulin prior to ANC, those with gestational diabetes were receiving the smallest dose and women with type 2 diabetes the largest dose, but the percentage increase in dose after ANC was approximately 80% for all types of diabetes (S5 Table).

### Neonatal glycaemic control

Glycaemic data were available for 713 of the 715 babies. The median (IQR) number of blood glucose measurements was 7 (4–10). Hypoglycaemia occurred in 46% (331/713) of babies, and was severe in 27% (193/713) and recurrent in 11% (77/713), while 6% (43/713) developed hyperglycaemia. Of the 331 babies who developed hypoglycaemia, 75% (247/331) did so within 2 hours of birth and 8% (25/331) after 24 hours.

Using univariate analysis, neonatal hypoglycaemia was associated with (a) maternal variables: type of diabetes; ethnicity; maternal year of birth; mode of birth; multiple pregnancy, and BMI; (b) ANC variables: time from ANC to birth; repeat course of ANC; number of doses of last course, and latest gestation ANC administered; and (c) neonatal variables: birth gestation; birthweight; birthweight centile; birthweight z-score, and sex (S6 Table).

Using multivariate analysis including variables that were significant on the univariate analysis, the odds of neonatal hypoglycaemia and severe hypoglycaemia were increased with pre-existing diabetes compared to gestational diabetes, caesarean section compared to spontaneous vaginal birth, a birth gestation of 35 weeks or more compared to < 35 weeks, an interval between ANC administration and birth of 12–48 hours compared to all other time intervals, female as opposed to male sex and increasing birthweight z-score (Table 3).

**Table 3:** Associations between maternal, antenatal corticosteroid, and neonatal variables and neonatal hypoglycaemia.

Maternal Variables	Neonatal Hypoglycaemia			Severe Hypoglycaemia			Recurrent Hypoglycaemia		
	OR	(95%CI)	p-value	OR	(95%CI)	p-value	OR	(95%CI)	p-value
**Type of diabetes**									
** GDM**	**Reference**								
Type-1 Diabetes	3.00	1.73–65.2	< 0.0001	3.57	2.07–6.17	< 0.0001	1.91	0.88–4.14	0.10
Type-2 Diabetes	1.62	1.04–2.55	0.034	1.81	1.20–3.22	0.007	1.89	0.99–3.63	0.055
**Ethnicity**									
** NZ European**	**Reference**								
Asian	0.82	0.48–1.34	0.45	0.57	0.31–1.07	0.08	1.02	0.42–2.44	0.97
Indian	1.11	0.66–1.86	0.70	0.76	0.42–1.39	0.38	1.35	0.58–3.16	0.49
Maori	0.76	0.42–1.37	0.36	0.63	0.33–1.23	0.18	0.80	0.28–2.65	0.62
Pacific	0.84	0.49–1.44	0.52	0.58	0.31–1.07	0.09	0.98	0.44–2.21	0.97
Other	0.97	0.52–1.83	0.94	0.65	0.32–1.35	0.25	1.17	0.43–3.21	0.76
**Mode of birth**									
** SVB**	**Reference**								
OVB	0.96	0.44–2.09	0.91	1.31	0.53–3.26	0.55	1.22	0.36–4.19	0.75
Elective Caesarean	2.44	1.60–3.78	< 0.0001	2.27	1.36–3.81	0.002	1.66	0.82–3.39	0.16
Emergency Caesarean	1.58	1.02–2.44	0.040	1.26	0.74–2.14	0.55	1.20	0.58–2.50	0.75
**Multiple pregnancy**									
** Singleton**	**Reference**								
Multiple	0.77	0.51–1.18	0.23	0.91	0.50–1.31	0.70	0.41	0.17–0.95	0.038
**BMI**									
** 20–24.9**	**Reference**								
< 20	0.84	0.35–2.21	0.72	1.46	0.53–4.09	0.46	1.81	0.45–7.30	0.40
25–29.9	0.96	0.62–1.51	0.87	1.39	0.82–2.36	0.18	1.50	0.68–3.13	0.31
≥ 30	1.34	0.87–2.07	0.19	1.71	1.03–2.86	0.038	2.21	1.04–4.73	0.04
**Birth year**									
** 2014–2016**	**Reference**								
2006–2009	0.76	0.50–1.17	0.22	0.92	0.57–1.48	0.73	0.33	0.17–0.64	0.001
2010–2013	0.90	0.61–1.33	0.60	0.91	0.58–1.42	0.68	0.53	0.30–0.93	0.03
**ANC Variables**									
**Number of doses in pregnancy**									
** 2 doses**	**Reference**								
1 dose	0.81	0.23–2.86	0.74	0.83	0.19–3.54	0.80	0.14	0.01–2.00	0.14
3 or more doses	0.76	0.27–2.11	0.60	0.56	0.17–1.81	0.33	0.21	0.02–2.1	0.20
**Time between doses last course**									
** 24 hours**	**Reference**								
12 hours	0.70	0.31–1.57	0.39	1.08	0.47–2.47	0.85	0.57	0.12–2.58	0.47
1 dose only	0.86	0.30–2.50	0.79	1.03	0.30–3.53	0.95	1.71	0.17–16.9	0.64
**Time between ANC administration and birth**									
** 12–48 hours**	**Reference**								
< 12hours	0.54	0.25–1.15	0.11	0.27	0.11–0.68	0.006	4.06	0.97–16.9	0.055
48 hours– 7 days	0.50	0.31–0.80	0.004	0.39	0.24–0.65	0.0003	1.44	0.65–3.17	0.36
≥ 8 days	0.41	0.26–0.65	0.0002	0.31	0.19–0.51	< 0.0001	1.32	0.60–2.87	0.48
**Latest gestation at ANC administration**									
** 24–34 weeks**	**Reference**								
≥ 35 weeks	1.74	0.97–3.10	0.059	1.61	0.78–3.31	0.19	1.74	0.60–5.04	0.30
**Neonatal Variables**									
**Birth gestation**									
**24**^**0**^**−34**^**6**^ **weeks**	**Reference**								
35^0^–36^6^ weeks	1.57	1.08–2.30	0.017	1.32	0.87–1.99	0.18	2.12	1.16–3.88	0.015
≥ 37 weeks	1.01	0.69–1.46	0.94	0.78	0.51–1.20	0.78	2.16	1.91–3.92	0.011
**Sex of the baby**									
** Male**	**Reference**								
Female	1.52	1.12–2.06	0.007	1.53	1.09–2.16	0.015	1.64	1.01–2.69	0.044
**Birthweight z-score**^**a**^	1.16	1.04–1.31	0.008	1.29	1.13–1.46	< 0.0001	1.28	1.08–1.51	0.003

ANC–Antenatal corticosteroids, BMI–Body mass index. GDM–Gestational diabetes, NZ–New Zealand, SVB–spontaneous vaginal birth, OVB–operative vaginal birth. Analyses are by multiple nominal logistic regression.

^a^ Unit odds ratio (OR) expressed as the change in the rate of the relevant neonatal glycaemic variable for each unit change of z-score.

The increased odds of recurrent hypoglycaemia were similar for type-1 and type-2 diabetes compared to gestational diabetes, but were statistically significant only for type-2 diabetes. The odds of recurrent hyperglycaemia were also increased for singleton compared to multiple pregnancies, maternal BMI ≥ 30 kg/m^2^, birth-year 2014–16, late preterm gestation at birth compared to early term or term, female compared to male sex and increasing birthweight z-score (Table 3).

### Association between maternal and neonatal glycaemic control

The relationships between maternal glycaemia after the last course of ANC and in the 24 hours before the birth and neonatal hypoglycaemia or severe hypoglycaemia were assessed for 644 and 634 mother-infant pairs respectively. For 233/626 (37%) women and 265/693 (38%) babies, 24 hours prior to birth was within 72 hours of the first dose of a course of ANC. The median (IQR) peak BGC in the 24 hours prior to birth was 6.8 (5.7–8.7) mmol/L, mean BGC was 5.5 (4.8–6.5) mmol/L and last BCG was 5.3 (4.5–6.3) mmol/L.

The odds of neonatal hypoglycaemia were increased in babies of mothers who had BGC above 7 and 8 mmol/L and who had a fasting BGC ≥ 5.5 mmol/L within 24 hours of birth. The odds also increased with increasing mean BGC and increasing last BGC prior to birth (Table 4). The odds of severe hypoglycaemia were higher but followed the same pattern of relationships with maternal glycaemia. There was no association between neonatal hypoglycaemia and maternal glycaemic control at the time of the last course of ANC administration except for an increase in odds of severe hypoglycaemia in babies whose mothers had BGC > 11 mmol/l (Table 4).

**Table 4:** Association between neonatal hypoglycaemia and measures of maternal glycaemic control within 24 hours of birth and after the last course of antenatal corticosteroids.

**Maternal glycaemia in the 24 hours before birth**									
**Mothers N = 634**		**Babies with hypoglycaemia N = 301**				**Babies with severe hypoglycaemia N = 179**			
	**N (%)**	**N (%)**^**a**^	**OR**^**b**^	**95% CI**	**P value**	**N (%)**^**a**^	**OR**^**b**^	**95% CI**	**P value**
**BGC > 7 mmol/L**	292 (46%)	155 (52%)	1.51	1.10–2.07	0.01	105 (72%)	2.00	1.41–2.85	< 0.0001
**BGC > 8 mmol/L**	201 (32%)	114 (38%)	1.72	1.22–2.40	0.002	82 (56%)	2.36	1.65–3.38	< 0.0001
**BGC > 10 mmol/L**	77 (12%)	41 (14%)	1.30	0.80–2.09	0.29	33 (23%)	2.06	1.29–3.41	0.003
**BGC > 11 mmol/L**	53 (8%)	28 (9%)	1.26	0.72–2.21	0.42	25 (17%)	2.46	1.39–4.35	0.002
**BGC out of range**^**c**^	415 (65%)	201 (67%)	1.10	0.79–1.54	0.53	128 (72%)	1.46	1.00–2.12	0.046
**Fasting BGC ≥ 5.5 mmol/L**	214 (34%)	122 (40%)	1.56	1.09–2.24	0.015	85 (58%)	1.80	1.24–2.67	0.002
**Peak BGC**		-	1.05	0.99–1.12	0.11	-	1.14	1.07–1.23	0.0001
**Mean BGC**		-	1.17	1.04–1.32	0.008	-	1.25	1.10–1.41	0.0005
**Last BGC before birth**		-	1.11	1.00–1.22	0.047	-	1.13	1.02–1.26	0.025
**Maternal glycaemia after the last course of ANC**									
**Mothers N = 640**		**Babies with hypoglycaemia Total = 300**				**Babies with severe hypoglycaemia Total = 179**			
	**N (%)**	**N (%)**^**a**^	**OR**^**b**^	**95% CI**	**P value**	**N (%)**^**a**^	**OR**^**b**^	**95% CI**	**P value**
**BGC > 7 mmol/L**	579 (90%)	270 (90%)	0.91	0.53–1.54	0.71	162 (91%)	1.01	0.57–1.86	0.98
**BGC > 8 mmol/L**	504 (78%)	239 (80%)	1.15	0.79–1.68	0.53	146 (82%)	1.31	0.85–2.04	0.22
**BGC > 10 mmol/L**	295 (46%)	143 (48%)	1.15	0.84–1.56	0.39	91 (51%)	1.32	0.93–1.86	0.11
**BGC > 11 mmol/L**	197 (31%)	100 (33%)	1.27	0.90–1.77	0.17	66 (37%)	1.47	1.02–2.12	0.036
**BGC Out of range**^**c**^	581 (90%)	270 (90%)	0.78	0.46–1.34	0.37	162 (91%)	0.88	0.49–1.60	0.66
**Fasting BGC ≥ 5.5 mmol/L**	516 (80%)	245 (83%)	1.08	0.71–1.66	0.72	147 (82%)	0.99	0.62–1.6	0.96
**Peak BGC**			1.01	0.96–1.07	0.98		1.05	0.97–1.12	0.95

OR, Odds ratio; BGC, Blood glucose concentration.

a. Only including babies whose mothers exceed the given threshold.

b. Women who do not exceed the specified threshold are the reference group for categorical variables. For continuous variables, the odds ratio is the increase in the rate of neonatal hypoglycaemia or severe hypoglycaemia for a change of 1 mmol/L in maternal blood glucose concentration.

c. Out of range, < 4 or > 7 mmol/L.

## Discussion

### Main findings

In this large cohort of women with DIP who received ANC, maternal hyperglycaemia was common. Over 90% of women developed hyperglycaemia > 7 mmol/L after an initial or repeat course of ANC, and over 50% developed hyperglycaemia at higher thresholds. Previous studies, which focused on the insulin dose required to maintain euglycaemia, have also reported clinically significant maternal hyperglycaemia after ANC administration in women with both gestational and pre-existing diabetes [5, 13] including when continuous blood glucose monitoring was used to assist with glycaemic management [26]. Although women with pre-existing diabetes exhibited poorer glycaemic control before and after ANC administration, a high proportion of women with gestational diabetes were also hyperglycaemic after ANC administration. The percentage increase in insulin dosage after ANC administration was similar for women with type-1, type-2 and gestational diabetes.

During an initial course of ANC, maternal BGC peaked approximately 9 hours after each dose of ANC and did not return to baseline for approximately 72 hours. As the drug, dose and number of doses in a course of ANC administered to this cohort was highly uniform, it was not possible to explore the associations between these variables and maternal and neonatal glycaemic control. However, in the multivariate analysis, maternal hyperglycaemia at the lowest threshold (7 mmol/L) was less likely following a schedule of two doses 12 hours apart than 24 hours apart, although there was no effect at higher thresholds. This was not due to women receiving ANC at 12 hour intervals giving birth before they had time to develop hyperglycaemia.

Both 12 and 24-hour interval schedules have been shown to decrease the complications of preterm birth [27]. Therefore, particularly in women birthing electively by either caesarean section or induction, for whom current recommendations are to administer ANC 48 hours prior to planned birth [7], future studies should explore whether it is possible to reduce the proportion of women with DIP giving birth while hyperglycaemic by increasing the time interval between ANC and birth and/or decreasing the time interval between doses.

A small group of women birthed soon after ANC administration and as a result received only one dose of ANC. These women were less likely to develop hyperglycaemia than women who did not birth until after two doses of ANC had been administered. However, it was not possible to determine if the association between ANC dose and glycaemic control was due to the single dose, or whether these women did not have time to reach peak hyperglycaemia before they gave birth.

Maternal hyperglycaemia within 24 hours of birth is associated with increased odds of approximately 1.5 for neonatal hypoglycaemia and > 2.0 for severe hypoglycaemia. Given that 30% of women birthed within 72 hours of commencement of ANC, a significant proportion of women are likely to be hyperglycaemic at the time they gave birth and their babies are at increased risk of neonatal hypoglycaemia. The association between maternal hyperglycaemia and neonatal hypoglycaemia is strengthened by the observation that maternal blood glucose concentration peaked from 12 to 72 hours after ANC administration and babies of women birthing from 12 hours to 48 hours after ANC administration were more likely to develop hypoglycaemia than those born before or after this period.

The reason for the increased odds of neonatal hypoglycaemia after elective caesarean section compared to vaginal birth cannot be identified from this study. However, as elective caesarean section was the most frequent mode of birth in this cohort and the use of ANC prior to elective caesarean is common [28] and increasing [29], this association is of concern.

The rate of severe maternal hyperglycaemia declined over the period of the study, potentially related to more active management of maternal glycaemic control after ANC administration latterly, despite a lack of consensus for glycaemic targets after ANC administration [7, 27, 30]. Even so, there was no significant change in the rate of neonatal hypoglycaemia over time. However, neonates born in the most recent epoch (2014 to 2016) were more likely to develop recurrent hypoglycaemia than those born in earlier epochs, even though maternal hyperglycaemia at higher thresholds (> 8 mmol/L) was less common. The last epoch coincided with the introduction of oral dextrose gel as an initial treatment for neonatal hypoglycaemia at this hospital. Oral dextrose gel has been shown to be a safe and effective method of treating neonatal hypoglycaemia, and did not increase the risk of recurrent hypoglycaemia in the original randomised trial [22]. It is possible that other aspects of neonatal care were also changing over this period, including improved screening protocols for babies at risk.

### Interpretation

Maternal hyperglycaemia is common in women with DIP after ANC administration, supporting current recommendations to monitor the BGC of all women with DIP receiving ANC [7]. Our findings suggest that the BGC targets for intrapartum management are commonly exceeded after ANC administration [27, 28]. This study suggests that future research should explore whether the rate of maternal hyperglycaemia after ANC administration may be decreased by alterations in the timing of ANC administration and the dose interval. There was insufficient evidence to assess the effect of the drug used on maternal hyperglycaemia.

Both neonatal hypoglycaemia and severe hypoglycaemia are common in babies of women with DIP who receive ANC, potentially placing these babies at risk of long-term cognitive impairment [17]. The risk of neonatal hypoglycaemia is more closely related to glycaemic control immediately prior to birth than to glycaemic control after ANC. Since hyperglycaemia after ANC administration resolves after approximately 72 hours, administration of ANC more than 72 hours before birth may reduce the risk of neonatal hypoglycaemia.

### Strengths and limitations

One limitation of this study is its retrospective nature, so that all variables were not consistently available for all women and babies, and BGC testing schedules were variable. Further, the consistency of the drug and schedule of ANC, and correlation between the number of doses in a course and the time from ANC administration to birth, limited our ability to fully assess the effect of ANC variables on maternal and neonatal glycaemia. The sample size was large and was from a single centre, providing consistency but potentially reducing generalisability to hospitals with different practices. Several thresholds for maternal hyperglycaemia were assessed along with other parameters of glycaemic control, and the consistent results across all parameters of maternal glycaemic control suggest the findings are robust.

## Conclusion

Maternal hyperglycaemia is common after ANC administration in women with DIP, lasts for approximately 72 hours and may be affected by the number of doses administered and the time between doses. Babies born to mothers with hyperglycaemia within 24 hours of birth are at increased risk of developing hypoglycaemia. Future studies should explore whether the risk of neonatal hypoglycaemia may be able to be reduced by tighter control of maternal glycaemia within 24 hours of birth in women with DIP.

## Supporting information

S1 Table Characteristics of the initial and last course of antenatal corticosteroids. (DOCX)

S2 Table Measures of maternal glycaemic control after antenatal corticosteroid administration and in the 24 hours prior to birth, and after the initial course of antenatal corticosteroids for women with type-1, type2 and gestational diabetes. (DOCX)

S3 Table Maternal hyperglycaemia at different thresholds after the initial, last and last repeat course of antenatal corticosteroids, and after the initial course of antenatal corticosteroids for women with type 1, type 2 and gestational diabetes. (DOCX)

S4 Table Multivariate analysis of the relationships between maternal characteristics and the rate of hyperglycaemia at different glucose thresholds. (DOCX)

S5 Table Maternal insulin administration before and after antenatal corticosteroid administration. (DOCX)

S6 Table Univariate analysis of the associations between maternal, neonatal and antenatal corticosteroid variables and neonatal hypoglycaemia. (DOCX)
