# Supplementary material for: Maternal and neonatal glycaemic control after antenatal corticosteroid administration in women with diabetes in pregnancy: A retrospective cohort study
Source: PLoS One. 2021 Feb 18;16(2):e0246175. doi: 10.1371/journal.pone.0246175 (PMC7891747; doi:10.1371/journal.pone.0246175)
Supplement: S1 Table — (DOCX) [file pone.0246175.s001.docx]

**S1 Table. Characteristics of the initial and last course of antenatal corticosteroid.**

| **Initial course of antenatal corticosteroid** | **All women with glycaemic data** | |
| --- | --- | --- |
|  | **N = 626^a^** | **%** |
| **Drug** |  |  |
| Betamethasone | 608 | 97 |
| **Schedule** |  |  |
| 1 dose | 58 | 9 |
| 2 doses - 12 hours | 33 | 6 |
| 2 doses - 24 hours | 535 | 85 |
| **Time from initial dose to birth** |  |  |
| 0 – 12 hours | 37 | 6 |
| > 12 – 48 hours | 93 | 15 |
| > 48 – 7 days | 159 | 25 |
| > 7 days | 337 | 54 |
| **Gestation at first dose (weeks)** |  |  |
| 24^0^ - 27^6^ | 127 | 26 |
| 28^0^ – 34^6^ | 392 | 63 |
| 35^0^ – 36^6^ | 96 | 15 |
| 37^0^ - 38^0^ | 11 | 2 |
| **Last course of antenatal corticosteroid** | **N = 626** | **%** |
| **Schedule** |  |  |
| 1 dose | 161 | 26 |
| 2 doses - 12 hours | 22 | 4 |
| 2 doses - 24 hours | 443 | 71 |
| **Repeat course of antenatal corticosteroid (ANC)** |  |  |
| Yes | 113 | 18 |
| 1 dose | 104 | 92 |
| 2 doses | 9 | 8 |
| **Total number of doses in pregnancy** |  |  |
| 1-2 | 513 | 82 |
| 3 | 62 | 16 |
| > 3 | 51 | 8 |
| **Gestation at first dose of last course of ANC (weeks)** |  |  |
| 24^0^ - 27^6^ | 60 | 10 |
| 28^0^ – 34^6^ | 422 | 67 |
| 35^0^ – 36^6^ | 92 | 15 |
| 37^0^ - 38^0^ | 52 | 8 |
| **Number of doses in 7 days before birth** |  |  |
| 0 | 257 | 41 |
| 1 | 121 | 19 |
| 2 | 248 | 40 |
| **Time from first dose of last course of ANC to birth** |  |  |
| 0 – 12 hours | 53 | 8 |
| 12 – 48 hours | 119 | 19 |
| 48 – 7 days | 187 | 30 |
| More than 7 days | 267 | 43 |

**^a^** Includes all women with glycaemia data after the initial or last course of ANC
