# Supplementary material for: Maternal and neonatal glycaemic control after antenatal corticosteroid administration in women with diabetes in pregnancy: A retrospective cohort study
Source: PLoS One. 2021 Feb 18;16(2):e0246175. doi: 10.1371/journal.pone.0246175 (PMC7891747; doi:10.1371/journal.pone.0246175)
Supplement: S2 Table — (DOCX) [file pone.0246175.s002.docx]

**S2 Table. Measures of maternal glycaemic control after antenatal corticosteroid administration and in the 24 hours prior to birth, and after the initial course of antenatal corticosteroids for women with type-1, type2 and gestational diabetes.**

|  | **Number of measurements per woman**  **(median, IQR)** | | **Peak BGC**  **(Median, Range)**  **(mmol/L)** | | **Measurements out of range, %**  **(median, IQR)** | | **Women with fasting BGC ≥ 5 .5 mmol/L**  **(n/N, %)** | | **Women with hypoglycaemia before ANC**  **(n/N, %)** | | | **Women with hypoglycaemia after ANC**  **(n/N, %)** | |
| --- | --- | --- | --- | --- | --- | --- | --- | --- | --- | --- | --- | --- | --- |
| **Initial course** | 17 | 7-30 | 10.1 | 4.5-27.3 | 37 | 24-54 | 462/538 | 86 | 87/403 | 22 | 156/556 | | 28 |
| **Last course** | 15 | 6-28 | 9.9 | 4.2-26 | 37 | 23-54 | 468/558 | 84 | 89/442 | 20 | 150/580 | | 26 |
| **Repeat course** | 11 | 3-26 | 9.2 | 4.2-27.3 | 31 | 15-50 | 27/110 | 75 | 14/86 | 16 | 24/118 | | 20 |
| **Prior to birth** | 5 | 3-7 | 6.8 | 3.4-23.0 | 25 | 0-50 | 200/450 | 44 | N/A | | N/A | | |

**Type of diabetes**

| **T1DM (N=77)** | 24 | 17-42 | 13.4 | 6.6-27.3 | 59 | 50-71 | 75/77 | 97 | 31/63 | 49 | 45/77 | 58 |
| --- | --- | --- | --- | --- | --- | --- | --- | --- | --- | --- | --- | --- |
| **T2DM**  **(N=119)** | 24 | 14-40 | 10.8 | 5.6-20.0 | 43 | 31-56 | 111/116 | 96 | 30/102 | 29 | 60/119 | 50 |
| **GDM**  **(N=360)** | 17 | 10-29 | 9.5 | 4.5-15.9 | 33 | 20-46 | 27/110 | 75 | 28/241 | 12 | 51/360 | 14 |

IQR, interquartile range; BGC, blood glucose concentration; ANC, antenatal corticosteroids; N/A, not applicable; out of range, < 4 or > 7 mmol/L, T1DM, type 1 diabetes; T2DM, type 2 diabetes; GDM, gestational diabetes.
