# Supplementary material for: Maternal and neonatal glycaemic control after antenatal corticosteroid administration in women with diabetes in pregnancy: A retrospective cohort study
Source: PLoS One. 2021 Feb 18;16(2):e0246175. doi: 10.1371/journal.pone.0246175 (PMC7891747; doi:10.1371/journal.pone.0246175)
Supplement: S3 Table — (DOCX) [file pone.0246175.s003.docx]

S3 Table. Maternal hyperglycaemia at different thresholds after the initial, last and last repeat course of antenatal corticosteroids, and after the initial course of antenatal corticosteroids for women with type 1, type 2 and gestational diabetes.

| **Blood glucose threshold** | **Maternal hyperglycaemia**  **(n, %)** | | | **Hyperglycaemia prior to ANC**  **(n, %)** | | | **Percent of hyperglycaemic measurements**  **(median, IQR)** | | | **Time from ANC to onset of hyperglycaemia (hours, median (IQR)** | | | **Time from onset to resolution of hyperglycaemia**  **(hours, median, IQR)** | | | **Duration of hyperglycaemia**  **(hours, median, IQR)** | |
| --- | --- | --- | --- | --- | --- | --- | --- | --- | --- | --- | --- | --- | --- | --- | --- | --- | --- |
| **Initial course** | | **N=554** | | **N=403** | |  | | | | | | | | | | | |
| 7 mmol/L | 512 | | 92 | 183 | 45 | 33% | | 19-48 | 9 | | 4-13 | 56 | | 39-117 | 43 | | 27-104 |
| 8 mmol/L | 458 | | 83 | 117 | 29 | 17% | | 6-31 | 10 | | 5-17 | 45 | | 34-99 | 32 | | 14-83 |
| 10 mmol/L | 286 | | 52 | 60 | 15 | 3% | | 0-11 | 13 | | 8-23 | 36 | | 19-59 | 17 | | 4-43 |
| 11 mmol/L | 190 | | 35 | 37 | 9 | 0% | | 0-5 | 13 | | 8-28 | 37 | | 19-59 | 14 | | 3-36 |
| **Last course** | | **N=579** | | **N=442** | |  | | | | | | | | | | | |
| 7 mmol/L | 528 | | 91 | 203 | 46 | 31% | | 17-50 | 8 | | 4-13 | 49 | | 35-105 | 38 | | 22-89 |
| 8 mmol/L | 462 | | 80 | 130 | 29 | 17% | | 5-32 | 9 | | 4-15 | 42 | | 28-80 | 27 | | 12-65 |
| 10 mmol/L | 271 | | 47 | 63 | 14 | 0% | | 0-10 | 12 | | 7-21 | 34 | | 18-50 | 12 | | 4-36 |
| 11 mmol/L | 183 | | 32 | 41 | 9 | 0% | | 0-5 | 13 | | 7-25 | 35 | | 17-50 | 12 | | 3-31 |
| **Repeat course** | | **N=107** | | **N=86** | |  | | | | | | | | | | | |
| 7 mmol/L | 98 | | 92 | 43 | 50 | 25% | | 11-42 | 8 | | 4-16 | 50 | | 25-137 | 17 | | 10-39 |
| 8 mmol/L | 80 | | 75 | 27 | 31 | 11% | | 3-26 | 7 | | 4-15 | 30 | | 20-111 | 15 | | 9-32 |
| 10 mmol/L | 38 | | 36 | 8 | 9 | 0% | | 0-5 | 11 | | 6-21 | 24 | | 14-101 | 8 | | 3-17 |
| 11 mmol/L | 28 | | 26 | 7 | 8 | 0% | | 0-3 | 11 | | 6-22 | 23 | | 13-105 | 5 | | 2-12 |

A.

**Type of diabetes**

| **T1DM** | | **N=77** | | **N=63** | |  | | | | | | | | |
| --- | --- | --- | --- | --- | --- | --- | --- | --- | --- | --- | --- | --- | --- | --- |
| 7 mmol/L | 76 | | 99 | 45 | 71 | 48% | 38-66 | 7 | 3-11 | 52 | | 35-110 | 45 | 26-104 |
| 8 mmol/L | 75 | | 97 | 37 | 59 | 37% | 29-51 | 8 | 3-12 | 57 | | 35-130 | 49 | 25-118 |
| 10 mmol/L | 68 | | 88 | 27 | 43 | 17% | 9-27 | 12 | 8-17 | 45 | | 33-101 | 30 | 19-82 |
| 11 mmol/L | 59 | | 77 | 20 | 32 | 10% | 1-19 | 14 | 9-31 | 42 | | 26-90 | 27 | 3-67 |
| **T2DM** | | **N=119** | | **N=102** | |  | | | | | | | | |
| 7 mmol/L | 113 | | 95 | 52 | 51 | 32% | 20-50 | 7 | 4-11 | 60 | | 41-144 | 51 | 29-129 |
| 8 mmol/L | 108 | | 91 | 34 | 33 | 20% | 10-31 | 9 | 4-14 | 49 | | 34-120 | 40 | 18-98 |
| 10 mmol/L | 76 | | 64 | 17 | 17 | 5% | 0-12 | 11 | 7-19 | 38 | | 19-91 | 26 | 5-50 |
| 11 mmol/L | 57 | | 48 | 11 | 11 | 0% | 0-7 | 11 | 7-19 | 37 | | 16-66 | 22 | 3-40 |
| **GDM** | | **N=360** | | **N=241** | | **N=359** | |  | | |  | |  | |
| 7 mmol/L | 325 | | 90 | 89 | 37 | 29% | 15-42 | 10 | 5-15 | 54 | | 39-115 | 42 | 26-96 |
| 8 mmol/L | 227 | | 77 | 44 | 18 | 13% | 3-25 | 12 | 5-21 | 44 | | 34-77 | 29 | 10-61 |
| 10 mmol/L | 144 | | 40 | 13 | 5 | 0% | 0-6 | 14 | 8-29 | 33 | | 17-44 | 6 | 3-27 |
| 11 mmol/L | 76 | | 21 | 5 | 2 | 0% | 0-0 | 16 | 6-30 | 42 | | 18-41 | 7 | 3-27 |

IQR, interquartile range; BGC, blood glucose concentration; ANC, antenatal corticosteroids; N, number of women with glycaemic measurements for that variable, T1DM, type 1 diabetes; T2DM, type 2 diabetes; GDM, gestational diabetes.
