# Supplementary material for: Maternal and neonatal glycaemic control after antenatal corticosteroid administration in women with diabetes in pregnancy: A retrospective cohort study
Source: PLoS One. 2021 Feb 18;16(2):e0246175. doi: 10.1371/journal.pone.0246175 (PMC7891747; doi:10.1371/journal.pone.0246175)
Supplement: S4 Table — (DOCX) [file pone.0246175.s004.docx]

S4 Table. Multivariate analysis of the relationships between maternal characteristics and the rate of hyperglycaemia at different glucose thresholds.

| **Multivariate Analysis^a^** | | | | | | | | | | | | | | | | | | | | | | | | | | | | | | | | |
| --- | --- | --- | --- | --- | --- | --- | --- | --- | --- | --- | --- | --- | --- | --- | --- | --- | --- | --- | --- | --- | --- | --- | --- | --- | --- | --- | --- | --- | --- | --- | --- | --- |
|  | 7 mmol/L | | | | | | | | 8 mmol/l | | | | | | | 10 mmol/l | | | | | | | | | 11 mmol/l | | | | | | | |
| **Ethnicity** | **OR** | | | **95%CI** | | **P value** | **OR** | | | **95%CI** | **P value** | | | | **OR** | | **95%CI** | | **P value** | | | | | | **OR** | **95%CI** | | | **P value** | | | |
| **NZ European** | | | **Reference** | | | | | | | | | | | | | | | | | | | | | | | | | | | | | |
| Asian | 1.47 | | | 0.54-3.96 | | 0.45 | | | 0.78 | 0.37-1.6 | | 0.88 | | | | 1.01 | | 0.54-1.91 | | 0.95 | | | | | 0.68 | | | 0.32-1.46 | | | | 0.32 |
| Māori | 1.26 | | | 0.33-4.38 | | 0.78 | | | 0.72 | 0.29-1.84 | | 0.50 | | | | 2.18 | | 1.04-4.52 | | 0.04 | | | | | 1.23 | | | 0.58-2.57 | | | | 0.59 |
| Pacifica | 0.82 | | | 0.28-2.43 | | 0.73 | | | 0.57 | 0.25-1.29 | | 0.18 | | | | 0.91 | | 0.47-1.73 | | 0.76 | | | | | 0.83 | | | 0.41-1.69 | | | | 0.62 |
| Indian | 2.69 | | | 0.84-8.5 | | 0.094 | | | 1.26 | 0.56-2.86 | | 0.57 | | | | 1.75 | | 0.95-3.26 | | 0.07 | | | | | 1.37 | | | 0.70-2.70 | | | | 0.35 |
| Other | 1.28 | | | 0.37-4.42 | | 0.69 | | | 0.80 | 0.31-2.07 | | 0.65 | | | | 1.39 | | 0.65-2.96 | | 0.39 | | | | | 1.28 | | | 0.58-2.82 | | | | 0.53 |
| **Birth year** | | | | | | | | | | | | | | | | | | | | | | | | | | | | | | | | |
| **2006 – 2009** | | | **Reference** | | | | | | | | | | | | | | | | | | | | | | | | | | | | | |
| 2010 - 2013 | | 0.63 | | 0.28-1.40 | | 0.26 | | 0.46 | | 0.24-0.85 | | | | 0.014 | 0.59 | | | 0.38-0.91 | | | | 0.017 | | 0.47 | | | | 0.29-0.74 | | | | 0.001 |
| 2014 - 2016 | | 0.82 | | 0.32-2.13 | | 0.69 | | 0.42 | | 0.21-0.81 | | | | 0.011 | 0.43 | | | 0.26-0.72 | | | | 0.001 | | 0.56 | | | | 0.33-0.94 | | | | 0.031 |
| **Maternal age** | | | | | | | | | | | | | | | | | | | | | | | | | | | | | | | | |
| **25 – 35** | | | **Reference** | | | | | | | | | | | | | | | | | | | | | | | | | | | | | |
| < 25 | | 0.71 | | 0.17-2.84 | | 0.63 | | 1.09 | | 0.36-3.3 | | | | 0.87 | 1.14 | | | 0.59-3.29 | | | | 0.74 | | 1.53 | | | | 0.65-3.60 | | | | 0.32 |
| > 35 | | 0.93 | | 0.46-1.88 | | 0.85 | | 0.77 | | 0.46-1.26 | | | | 0.30 | 0.82 | | | 0.55-1.21 | | | | 0.33 | | 0.83 | | | | 0.54-1.27 | | | | 0.41 |
| **Parity** | | | | | | | | | | | | | | | | | | | | | | | | | | | | | | | | |
| **0** | | | **Reference** | | | | | | | | | | | | | | | | | | | | | | | | | | | | | |
| 1 | | 0.77 | | 0.36-1.65 | | 0.51 | | 1.19 | | 0.68-2.10 | | | | 0.53 | 0.96 | | | 0.61-1.52 | | | | 0.87 | | 0.87 | | | | 0.53-1.44 | | | | 0.61 |
| > 1 | | 1.49 | | 0.58-3.82 | | 0.40 | | 1.77 | | 0.92-3.4 | | | | 0.087 | 1.04 | | | 0.63-1.73 | | | | 0.63 | | 1.11 | | | | 0.65-1.91 | | | | 0.69 |
| **BMI** | | | | | | | | | | | | | | | | | | | | | | | | | | | | | | | | |
| **20 – 24.9** | | | **Reference** | | | | | | | | | | | | | | | | | | | | | | | | | | | | | |
| < 20 | | Inestimable | | |  | | | 0.85 | | 0.26-2.76 | | | 0.79 | | | 1.79 | | 0.63-5.03 | | | 0.27 | | | | 1.26 | | 0.39-4.12 | | | 0.69 | | |
| 25 – 29 | | Inestimable | | |  | | | 0.87 | | 0.44-1.71 | | | 0.69 | | | 1.50 | | 0.86-2.59 | | | 0.15 | | | | 1.27 | | 0.70-2.31 | | | 0.43 | | |
| > 30 | | Inestimable | | |  | | | 1.01 | | 0.51-1.96 | | | 0.98 | | | 1.71 | | 0.99-2.94 | | | 0.53 | | | | 1.18 | | 0.65-2.12 | | | 0.58 | | |
| **Multiple pregnancy** | | | | | | | | | | | | | | | | | | | | | | | | | | | | | | | | |
| **Singleton** | | | **Reference** | | | | | | | | | | | | | | | | | | | | | | | | | | | | | |
| Multiple | | 0.53 | | 0.22-1.28 | | 0.16 | | 0.47 | | 0.24-0.91 | | | 0.027 | | | 0.81 | | 0.44-1.51 | | | | | 0.52 | | 0.81 | | | 0.40-1.65 | | | 0.58 | |
| **Type of diabetes** | | | | | | | | | | | | | | | | | | | | | | | | | | | | | | | | |
| **GDM** | | | **Reference** | | | | | | | | | | | | | | | | | | | | | | | | | | | | | |
| Type-1 | | 12.4 | | 1.56-98.0 | | 0.017 | | 21.0 | | 2.77-159 | | | 0.003 | | 15.2 | | | 6.54-35.1 | | | | < 0.0001 | | | 13.0 | | | 6.57-25.7 | | < 0.0001 | | |
| Type-2 | | 1.95 | | 0.7-5.25 | | 0.18 | | 2.88 | | 1.38-6.02 | | | 0.005 | | 2.31 | | | 1.40-3.82 | | | | 0.001 | | | 3.00 | | | 1.78-5.05 | | < 0.0001 | | |

^a^ Multivariate analysis included all variables in Table S1.

OR, odds ratio; 95%CI, 95% confidence intervals; NZ, New Zealand; BMI, body mass index; GDM, gestational diabetes mellitus.
