# Supplementary material for: Maternal and neonatal glycaemic control after antenatal corticosteroid administration in women with diabetes in pregnancy: A retrospective cohort study
Source: PLoS One. 2021 Feb 18;16(2):e0246175. doi: 10.1371/journal.pone.0246175 (PMC7891747; doi:10.1371/journal.pone.0246175)
Supplement: S5 Table — (DOCX) [file pone.0246175.s005.docx]

**S5 Table. Maternal insulin administration before and after antenatal corticosteroid**

**administration.**

|  | **Insulin Prior to ANC^a^**  **(n/N %)** | | **Insulin After ANC^a^**  **(n/N %)** | | **Median (IQR) Insulin Dose Prior to ANC**  **(Median, IQR)** | | **Median (IQR) Insulin Dose After ANC**  **(Median, IQR)** | | **Percent change in Insulin Dose**  **(Median, IQR)** | | |
| --- | --- | --- | --- | --- | --- | --- | --- | --- | --- | --- | --- |
| **Type I Diabetes** | 78/78 | 100 | 78/78 | 100 | 74 | 49-95 | 128 | 104-170 | 78 | 41-120 |  |
| **Type 2 Diabetes** | 108/114 | 95 | 111/114 | 97 | 127 | 72-191 | 202 | 126-308 | 80 | 45-97 |  |
| **Gestational Diabetes** | 159/394 | 40 | 259/390 | 66 | 53 | 28-86 | 95 | 56-130 | 80 | 44-117 |  |

Only women with insulin data available were included. ^a^ Insulin data missing before ANC for 30 with GDM and 10 with T2DM, after ANC for 34 with GDM and 10 with T2DM. Insulin doses are total units per day.
