# Supplementary material for: Maternal and neonatal glycaemic control after antenatal corticosteroid administration in women with diabetes in pregnancy: A retrospective cohort study
Source: PLoS One. 2021 Feb 18;16(2):e0246175. doi: 10.1371/journal.pone.0246175 (PMC7891747; doi:10.1371/journal.pone.0246175)
Supplement: S6 Table — (DOCX) [file pone.0246175.s006.docx]

**S6 Table. Univariate analysis of the associations between maternal, neonatal and antenatal corticosteroid variables and neonatal hypoglycaemia.**

|  |  | **Neonatal hypoglycaemia**  **OR, 95%CI, p-value** | | | | | **Severe neonatal hypoglycaemia**  **OR, 95%CI, p-value** | | | **Recurrent hypoglycaemia**  **OR, 95%CI, p-value** | | |  |
| --- | --- | --- | --- | --- | --- | --- | --- | --- | --- | --- | --- | --- | --- |
| **Maternal Variables** | | | | | | | | | | | | |  |
| **Type of diabetes** | | | | | | | | | | | | |  |
| **GDM** | | **Reference** | |  |  |  | |  |  | |  |  |  |
| Type-1 Diabetes | | 3.07 | 1.91-5.05 | < 0.0001 | 4.09 | 2.55-6.59 | | < 0.0001 | 1.90 | | 0.94-3.62 | 0.07 |  |
| Type 2 Diabetes | | 1.73 | 1.17-2.56 | 0.006 | 2.04 | 1.33-3.11 | | 0.001 | 2.22 | | 1.25-3.85 | 0.007 |  |
| **Maternal ethnicity** | | | | | | | | | | | | |  |
| **NZ European** | | **Reference** | |  |  |  | |  |  | |  |  |  |
| Asian | | 0.52 | 0.33-0.81 | 0.004 | 0.34 | 0.20-0.58 | | < 0.0001 | 0.75 | | 0.34-1.62 | 0.46 |  |
| Indian | | 0.71 | 0.44-1.12 | 0.14 | 0.47 | 0.28-0.80 | | 0.0047 | 0.96 | | 0.45-2.06 | 0.93 |  |
| Maori | | 0.76 | 0.45-1.3 | 0.32 | 0.73 | 0.42-1.29 | | 0.28 | 1.10 | | 0.47-2.53 | 0.82 |  |
| Pacific | | 0.80 | 0.51-1.26 | 0.34 | 0.62 | 0.38-1.02 | | 0.059 | 1.47 | | 0.75-2.89 | 0.25 |  |
| Other | | 0.70 | 0.39-1.27 | 0.25 | 0.53 | 0.27-1.05 | | 0.061 | 0.98 | | 0.37-2.58 | 0.97 |  |
| **Maternal age** | | | | | | | | | | | | |  |
| 25-34 years | | **Reference** | |  |  |  | |  |  | |  |  |  |
| < 25 years | | 0.79 | 0.41-1.51 | 0.48 | 0.75 | 0.36-1.59 | | 0.46 | 1.30 | | 0.51-3.28 | 0.57 |  |
| ≥ 35 years | | 1.12 | 0.82-1.52 | 0.47 | 0.88 | 0.62-1.25 | | 0.49 | 0.97 | | 0.59-1.59 | 0.90 |  |
| **Parity** | | | | | | | | | | | | |  |
| **0** | | **Reference** | |  |  |  | |  |  | |  |  |  |
| 1 | | 0.95 | 0.67-1.35 | 0.95 | 0.88 | 0.58-1.32 | | 0.55 | 0.72 | | 0.39-1.27 | 0.26 |  |
| > 1 | | 1.15 | 0.80-1.65 | 0.44 | 1.23 | 0.82-1.83 | | 0.31 | 0.88 | | 0.49-1.54 | 0.26 |  |
| **Year of birth** | | | | | | | | | | | | |  |
| **2006-2009** | | **Reference** | | |  | | | |  | | | |  |
| 2010-2013 | | 1.14 | 0.81-1.62 | 0.45 | 0.98 | 0.66-1.46 | | 0.94 | 1.76 | | 1.00-3.09 | 0.049 |  |
| 2013-2016 | | 1.24 | 0.84-1.84 | 0.27 | 1.06 | 0.68-1.64 | | 0.77 | 2.09 | | 1.46-5.73 | 0.002 |  |
| **Mode of birth** | | | | | | | | | | | | |  |
| **SVB** | | **Reference** | |  |  |  | |  |  | |  |  |  |
| OVB | | 0.86 | 0.39-1.81 | 0.71 | 1.21 | 0.48-2.82 | | 0.66 | 1.39 | | 0.37-4.22 | 0.59 |  |
| Elective Caesarean | | 2.36 | 1.58-3.54 | < 0.0001 | 2.51 | 1.58-4.06 | | < 0.0001 | 1.82 | | 0.95-3.68 | 0.068 |  |
| Emergency Caesarean | | 1.65 | 1.10-2.48 | 0.015 | 1.53 | 0.95-2.53 | | 0.080 | 1.34 | | 0.67-2.77 | 0.41 |  |
| **Multiple pregnancy** | | | | | | | | | | | | |  |
| **Singleton** | | **Reference** | |  |  |  | |  |  | |  |  |  |
| Multiple | | 0.87 | 0.59-1.26 | 0.47 | 0.95 | 0.61-1.45 | | 0.83 | 2.4 | | 1.02-5.61 | 0.044 |  |
| **Maternal BMI (kg/m^2^)** | | | | | | | | | | | | |  |
| **20-24.9** | | **Reference** | | |  | | | |  | | | |  |
| < 20 | | 0.73 | 0.30-1.70 | 0.48 | 1.17 | 0.40-2.94 | | 0.75 | 2.1 | | 0.45-7.24 | 0.30 |  |
| 25 – 30 | | 1.18 | 0.78-1.78 | 0.42 | 1.58 | 0.98-2.54 | | 0.058 | 1.91 | | 0.90-4.19 | 0.09 |  |
| > 30 | | 1.43 | 1.00-2.04 | 0.047 | 1.76 | 1.17-2.69 | | 0.006 | 2.5 | | 1.16-5.42 | 0.018 |  |
| **ANC Variables** | | | | | | | | | | | | |  |
| **Time between last ANC administration and birth** | | | | | | | | | | | | |  |
| **12-48 hours** | | **Reference** | |  |  |  | |  |  | |  |  |  |
| < 12 hours | | 0.45 | 0.24-0.83 | 0.01 | 0.24 | 0.10-0.50 | | < 0.0001 | 1.69 | | 0.58-4.66 | 0.32 |  |
| 48 hours – 7 days | | 0.56 | 0.36-0.87 | 0.01 | 0.42 | 0.26-0.66 | | 0.0002 | 1.85 | | 0.89-4.15 | 0.09 |  |
| ≥ 8 days | | 0.44 | 0.29-0.67 | 0.0001 | 0.32 | 0.20-0.49 | | < 0.0001 | 1.64 | | 0.81-3.59 | 0.18 |  |
| **Repeat Course** | | | | | | | | | | | | |  |
| **No** | | **Reference** | |  |  |  | |  |  | |  |  |  |
| Yes | | 0.73 | 0.49-1.08 | 0.12 | 0.65 | 0.40-1.02 | | 0.063 | 0.46 | | 0.21-1.00 | 0.050 |  |
| **Time between doses Last Course** | | | | | | | | | | | | |  |
| **24 hours** | | **Reference** | |  |  |  | |  |  | |  |  |  |
| 12 hours | | 1.09 | 0.50-2.33 | 0.82 | 1.90 | 0.87-4.12 | | 0.10 | 0.52 | | 0.12-2.24 | 0.38 |  |
| **Number of doses of last course** | | | | | | | | | | | | |  |
| **2 doses** | | **Reference** | |  |  |  | |  |  | |  |  |  |
| 1 dose | | 0.75 | 0.53-1.06 | 0.10 | 0.68 | 0.34-1.01 | | 0.060 | 0.53 | | 0.27-0.95 | 0.035 |  |
| **Latest gestation at ANC administration** | | | | | | | | | | | | |  |
| **24-34 weeks** | | **Reference** | |  |  |  | |  |  | |  |  |  |
| ≥ 35 weeks | | 2.35 | 1.6-3.4 | < 0.0001 | 2.3 | 1.58-3.35 | | < 0.0001 | 2.11 | | 1.26-3.47 | 0.005 |  |
| **Neonatal Variables** | | | | | | | | | | | | |  |
| **Birth gestation** | | | | | | | | | | | | |  |
| **35 – 36 weeks** | | **Reference** | |  |  |  | |  |  | |  |  |  |
| 24-34 weeks | | 0.57 | 0.39-0.83 | 0.004 | 0.65 | 0.43-0.96 | | 0.034 | 0.39 | | 0.22-0.71 | 0.002 |  |
| ≥ 37 weeks | | 0.59 | 0.41-0.96 | 0.03 | 0.57 | 0.36-0.91 | | 0.019 | 0.95 | | 0.53-1.70 | 0.87 |  |
| **Neonatal birthweight** | | | | | | | | | | | | |  |
| **> 4000 g** | | **Reference** | |  |  |  | |  |  | |  |  |  |
| < 1500 g | | 0.27 | 0.12-0.55 | 0.0003 | 0.28 | 0.09-0.44 | | < 0.0001 | 0.17 | | 0.06-0.45 | 0.0004 |  |
| 1500-2499 | | 0.50 | 0.25-0.95 | 0.035 | 0.42 | 0.22-0.78 | | 0.008 | 0.20 | | 0.09-0.45 | 0.0002 |  |
| 2500-4000 | | 0.45 | 0.23-0.86 | 0.016 | 0.32 | 0.17-0.61 | | 0.0007 | 0.37 | | 0.18-0.80 | 0.013 |  |
| **Birthweight centile** | | | | | | | | | | | | |  |
| **10 – 90^th^ centile** | | **Reference** | | |  | | | |  | | | |  |
| < 10^th^ Centile | | 1.57 | 0.99-2.52 | 0.055 | 1.48 | 0.86-2.46 | | 0.14 | 1.63 | | 0.74-3.31 | 0.74 |  |
| > 90^th^ centile | | 2.08 | 1.42-3.06 | 0.0002 | 3.1 | 2.07-4.62 | | < 0.0001 | 3.21 | | 1.88-5.43 | < 0.0001 |  |
| **Sex of the baby** |  |  |  |  |  |  | |  |  | |  |  |  |
| **Male** | | **Reference** | |  |  |  | |  |  | |  |  |  |
| Female | | 1.50 | 1.11-2.02 | 0.007 | 1.49 | 1.07-2.08 | | 0.018 | 1.72 | | 1.07-2.79 | 0.025 |  |
| **Birthweight Z-score^a^** | | 1.19 | 1.06-1.33 | 0.002 | 1.29 | 1.15-1.47 | | < 0.0001 | 1.34 | | 1.14-1.57 | 0.0003 |  |

OR, Odds ratio; ANC, Antenatal corticosteroids; BMI, Body mass index; GDM, Gestational diabetes; NZ, New Zealand; SVB, spontaneous vaginal birth; OVB, operative vaginal birth; BMI, Body mass index; GDM, Gestational diabetes.

^a^ Unit odds ratio.
